# Identification of diagnostic biomarkers and molecular subtype analysis associated with m6A in Tuberculosis immunopathology using machine learning

**DOI:** 10.1038/s41598-024-81790-4

**Published:** 2024-12-02

**Authors:** Shoupeng Ding, Jinghua Gao, Chunxiao Huang, Yuyang Zhou, Yimei Yang, Zihan Cai

**Affiliations:** 1 Department of Medical Laboratory, Siyang Hospital, Siyang County, 237000 Jiangsu Province China; 2 Department of Laboratory Medicine, Gutian County Hospital, Gutian, 352200 China; 3 Department of Microbiology and Immunology, School of Basic Medical Sciences, Dali University, Dali, 671000 China; 4 Chuxiong Yi Autonomous Prefecture People’s Hospital, Chuxiong, 675000 China

**Keywords:** Tuberculosis, m6A, Bioinformatics, Subtyping, Risk model, Biotechnology, Computational biology and bioinformatics, Microbiology, Molecular biology

## Abstract

Tuberculosis (TB), ranking just below COVID-19 in global mortality, is a highly complex infectious disease involving intricate immunological molecules, diverse signaling pathways, and multifaceted immune processes. N6-methyladenosine (m6A), a critical epigenetic modification, regulates various immune-metabolic and pathological pathways, though its precise role in TB pathogenesis remains largely unexplored. This study aims to identify m6A-associated genes implicated in TB, elucidate their mechanistic contributions, and evaluate their potential as diagnostic biomarkers and tools for molecular subtyping. Using TB-related datasets from the GEO database, this study identified differentially expressed genes associated with m6A modification. We applied four machine learning algorithms—Random Forest, Support Vector Machine, Extreme Gradient Boosting, and Generalized Linear Model—to construct diagnostic models focusing on m6A regulatory genes. The Random Forest algorithm was selected as the optimal model based on performance metrics (area under the curve [AUC] = 1.0, *p* < 0.01), and a clinical predictive model was developed based on these critical genes. Patients were stratified into distinct subtypes according to m6A gene expression profiles, followed by immune infiltration analysis across subtypes. Additionally, Gene Ontology (GO) and Kyoto Encyclopedia of Genes and Genomes (KEGG) pathway enrichment analyses elucidated the biological functions and pathways associated with the identified genes. Quantitative real-time PCR (RT-qPCR) was used to validate the expression of key m6A regulatory genes. Analysis of the GSE83456 dataset revealed four differentially expressed m6A-related genes—YTHDF1, HNRNPC, LRPPRC, and ELAVL1—identified as critical m6A regulators in TB through the Random Forest model. The diagnostic significance of these genes was further supported by a nomogram, achieving a high predictive accuracy (95% confidence interval [CI]: 0.87–0.94). Consensus clustering classified patients into two m6A subtypes with distinct immune profiles, as principal component analysis (PCA) showed significantly higher m6A scores in Group A than in Group B (*p* < 0.05). Immune infiltration analysis highlighted significant correlations between key m6A genes and specific immune cell infiltration patterns across subtypes. This study highlights the potential of key m6A regulatory genes as diagnostic biomarkers and immunotherapy targets for TB, supporting their role in TB pathogenesis. Future research should aim to further validate these findings across diverse cohorts to enhance their clinical applicability.

**Supplementary Information:**

The online version contains supplementary material available at 10.1038/s41598-024-81790-4.

## Competing Interests

The authors declare they have no conflict of interest.

## Introduction

Tuberculosis (TB) is a highly contagious chronic disease caused by *Mycobacterium tuberculosis* (MTB). This disease poses significant challenges in terms of diagnosis and drug resistance^1,2^. Although MTB can infect multiple organs, pulmonary infection is its most common manifestation. The clinical symptoms of TB vary widely, ranging from asymptomatic latent infection to severe, life-threatening disease^3^. Currently, isoniazid is the primary drug used in TB treatment. However, it is associated with a high risk of hepatotoxicity, with 5-33% of patients experiencing drug-induced liver injury during the course of treatment^4^. This hepatotoxicity not only reduces the therapeutic efficacy and cure rate of TB but also significantly increases patient mortality risk. Consequently, there is an urgent need to develop safer and more effective alternative therapies to reduce treatment risks and improve efficacy.

In recent years, research has increasingly focused on the complex pathogenesis of TB. Studies suggest that TB develops as a result of interactions among MTB, host genetic factors, and environmental factors^5^. Based on this understanding, identifying early diagnostic biomarkers for TB and methods to detect at-risk populations are essential for effective disease control^6^. From a genetic perspective, researchers have started to investigate the potential role of RNA m6A modification in tuberculosis. m6A is a crucial RNA modification that regulates gene expression, cellular differentiation, and immune response. It is particularly significant in inflammatory response, antiviral immunity, and tumor regulation^7,8^. However, the specific mechanism of m6A modification in bacterial infections, especially in MTB infections, remains unclear. Most existing studies on m6A focus on immunomodulation in viral infections and cancers^9–11^. In contrast, few studies address its role in bacterial infections, and there is a notable lack of phased analysis across different pathological stages.

Studies have found that ESXB, a protein secreted by MTB, inhibits m6A modification and reduces the mRNA stability of genes associated with the anti-tuberculosis response^12,13^. Additionally, the interaction between ESXB and METTL14, a key factor in m6A modification, has been suggested as a potential target for TB therapy^14^. These findings indicate that m6A methylation may play a critical role in the pathogenesis of TB. m6A modification-related genes may be key regulators in the development and progression of the disease.

In this study, we systematically explored the dynamic changes of m6A modification across different pathological stages of tuberculosis. This was achieved for the first time by combining large-scale data analysis with experimental validation. We identified a novel set of m6A-targeted genes. The expression patterns of these genes are closely related to TB progression and prognosis, providing a fresh perspective on the role of m6A modification in TB.

Beyond identifying m6A-targeted genes associated with TB, we also analyzed the specific regulatory roles of these genes in the TB immune microenvironment. By conducting stage-specific sample analysis and immune cell-specific analysis, we uncovered the dynamic features of m6A modifications in regulating the immune microenvironment throughout the TB disease course. Compared to previous studies, our work fills a critical gap in understanding the association between m6A modifications and different pathological stages of TB. This study provides foundational data that supports future molecular diagnostics and the development of therapeutic strategies for TB.

## Materials and methods

### Data collection and processing

For data collection, we obtained the GSE83456 tuberculosis dataset from the GEO database, which includes peripheral blood samples from 45 tuberculosis (TB) patients and 61 healthy controls. The sequencing platform used was GPL10558 (Illumina HumanHT-12 V4.0 expression chip). To ensure comparability across samples, we normalized the expression matrix using the “normalizeBetweenArrays” function in the R package “limma.”

**Fig. 1:**
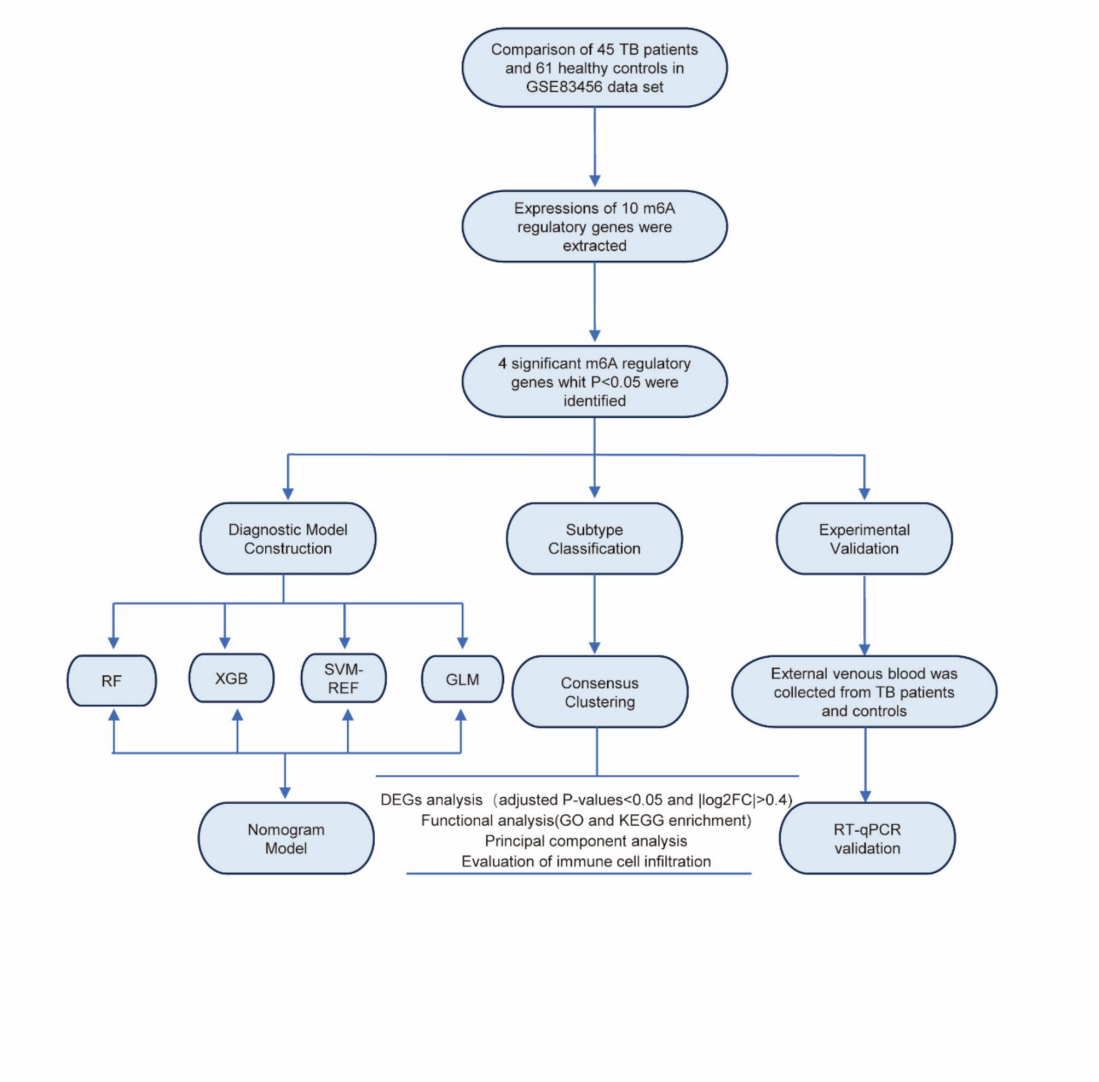
Experimental flow.

To increase the transparency of data preprocessing, we provided a detailed description of our handling of missing values and outliers. Missing values were imputed using a mean-filling method, and expression data were normalized by Z-scores to reduce variability across samples. Additionally, quality control checks were conducted on the data before standardization to ensure sample completeness and data consistency.The experimental design and workflow are depicted in Fig. 1.

### Identification of differentially expressed m6a regulatory genes in TB patients

Building on existing research into m6A modifications, we concentrated on 29 m6A-related genes, comprising 12 writer genes (METTL3, METTL14, METTL16, WTAP, VIRMA, ZC3H13, RBM15, RBM15B, CBLL1, YWHAG, TRA2A, CAPRIN1), 15 reader genes (YTHDC1, YTHDC2, YTHDF1, YTHDF2, YTHDF3, HNRNPC, FMR1, LRPPRC, HNRNPA2B1, IGFBP1, IGFBP2, IGFBP3, RBMX, ELAVL1, IGF2BP1), and 2 eraser genes (FTO, ALKBH5)^12^. We extracted the expression levels of these m6A-related genes using the “limma” package in R, and differential expression between TB patients and healthy controls was assessed via the Wilcoxon test. Genes with p-values < 0.05 were considered statistically significant. The chromosomal locations of these m6A-related genes were subsequently visualized using the “RCircos” package in R.

### Machine learning-based selection of key m6A regulatory genes

To identify key m6A-regulated genes associated with tuberculosis (TB), we constructed four machine learning models: Random Forest (RF), Support Vector Machine (SVM), Extreme Gradient Boosting (XGB), and Generalized Linear Model (GLM). We utilized the “RandomForest,” “kernlab,” “xgboost,” “DALEX,” and “caret” packages in R. To select the optimal model and identify the m6A-regulated genes with the most significant predictive effect, we plotted the residual and inverse cumulative distribution curves of differentially expressed m6A genes. All four machine learning algorithms were executed with the seed set to “123” to ensure reproducibility.

To ensure transparency and reproducibility, the following key parameters and settings were applied for each model. (1) Random Forest (RF): In the RF model, we set the number of trees (n_estimators) to 500 and the maximum depth (max_depth) to 10, balancing model complexity with computational efficiency. We used the Gini impurity as the criterion for node splitting and applied 5-fold cross-validation to evaluate model performance.(2) Support Vector Machine (SVM): The SVM model employed a Radial Basis Function (RBF) kernel, with the penalty parameter *C* set to 1.0 and γ set to 0.01. We optimized model performance using Grid Search and evaluated its effectiveness through 5-fold cross-validation. (3) Extreme Gradient Boosting (XGB): In the XGB model, we set the learning rate to 0.1, the maximum depth to 6, and the number of trees (n_estimators) to 100. Early stopping was used to prevent overfitting, and the model’s performance was evaluated with 5-fold cross-validation. (4) Generalized Linear Model (GLM): For the GLM model, we applied Lasso regularization to avoid overfitting. The regularization parameter αα was set to 0.1, and model selection was based on the Akaike Information Criterion (AIC).

Additionally, we used the Feature Importance Score (FIS) to assess the predictive value of each gene during feature selection. Genes with an FIS greater than 5 were considered as candidate genes, and the top-ranked genes based on FIS were ultimately selected as key m6A-regulated genes.

### Construction of a clinical prediction model

The identified key m6A regulatory genes were integrated using the “datadist” function from the “rms” package in R, after which a clinical prediction model was developed using the “lrm” function. The prediction model was visualized with the “nomogram” package. The model’s performance underwent rigorous evaluation, including discrimination assessed by the C-index, consistency evaluated through calibration curves, and clinical utility measured via decision and net benefit curves.

### Subtype classification of TB patients based on key m6A regulatory genes

We utilized the “ConsensusClusterPlus” package in R to cluster TB patient samples based on key m6A regulatory genes. The parameters were optimized as follows: “maxK"=6, “reps"=50, “pItem"=0.8, “pFeature"=1, “clusterAlg"="pam”, and “distance"="euclidean.” TB patients were categorized into six distinct subgroups. The optimal number of clusters was determined by the Calinski criterion and subgroup correlations. The distribution of samples across subgroups was visualized using the “Rtsne” package. The expression differences of key m6A regulatory genes among the subgroups were statistically evaluated using the Kruskal-Wallis test.

### Identification of differential genes between m6A subtypes and GO/KEGG enrichment analysis

Differentially expressed genes between m6A subtypes were identified using the “limma” package in R, with a stringent cutoff of |log2FC| > 0.4 and *p* < 0.05. Subsequently, the “clusterProfiler” package was utilized to perform GO and KEGG enrichment analyses to elucidate the potential mechanisms underlying these differential genes^15^.

### Calculation of m6A scores

Principal component analysis (PCA) was used to calculate m6A scores for TB samples, providing a quantitative measure of m6A subtype scores. The m6A score was computed using the formula: m6A score = PC1_i, where PC1 represents the first principal component, and i corresponds to the significant expression of m6A genes.

### Evaluation of immune cell infiltration

For immune cell infiltration analysis, we applied the single-sample gene set enrichment analysis (ssGSEA) method to calculate the infiltration scores of various immune cell types in each TB sample. First, we analyzed the expression level of each gene using ssGSEA, extracting the expression levels of key m6A-regulated genes from the dataset. To enhance the reliability of the analysis, we documented the gene sets and normalization steps applied in the ssGSEA analysis.

To further strengthen the scientific validity and reproducibility of our analysis, we introduced a threshold setting and applied a correction step in the immune infiltration analysis to ensure that the calculated immune cell abundances accurately reflected the actual state of each sample. Additionally, we performed statistical analyses on the abundance distributions of different immune cell types to evaluate the variation in immune components within TB samples.

### Experimental validation of key m6A gene expression levels

Peripheral blood mononuclear cells (PBMCs) from MTB-infected patients and healthy controls were collected, and serum heel cells were isolated by centrifugation at 4000 rpm. All procedures were in accordance with the Declaration of Helsinki. Ethical approval for this study was obtained from the Ethics Committee of Siyang Hospital. Total RNA was extracted using the Total RNA Extraction Kit (TIANGEN Biotech Co., Ltd., Beijing, China), following the manufacturer’s instructions. Reverse transcription was performed using the cDNA First Strand Synthesis Kit (TIANGEN). Real-time fluorescence quantitative PCR was conducted using the StepOne qPCR detection system, with qPCR primers listed in Table 1.

**Table 1:** Sequences of primers used quantitative real-time PCR (RT-qPCR).

Gene	Forward primer (5’ to 3’)	Reverse primer (5’ to 3’)
β-actin	GGCTGTATTCCCCTCCATCG	CCAGTTGGTAACAATGCCATGT
HNRNPC	CCCTTCTCCGTCCCCTCTAC	CCCGAGCAATAGGAGGAGGA
ELAVL1	GGGTGACATCGGGAGAACG	CTGAACAGGCTTCGTAACTCAT
LRPPRC	CGGAGGACTACTGAGCCCA	AGCGGCAGGTATCATTAAAAACT
YTHDF1	ATACCTCACCACCTACGGACA	GTGCTGATAGATGTTGTTCCCC

### Statistical analyses

Statistical analysis of continuous variables between the two groups was conducted using the non-parametric Wilcoxon rank-sum test, with *p* < 0.05 considered statistically significant. All analyses were performed using R software (version 4.3.2) and Prism 10 (GraphPad Software, USA).

## Results

### Identification of differentially expressed m6A genes in tuberculosis

**Fig. 2:**
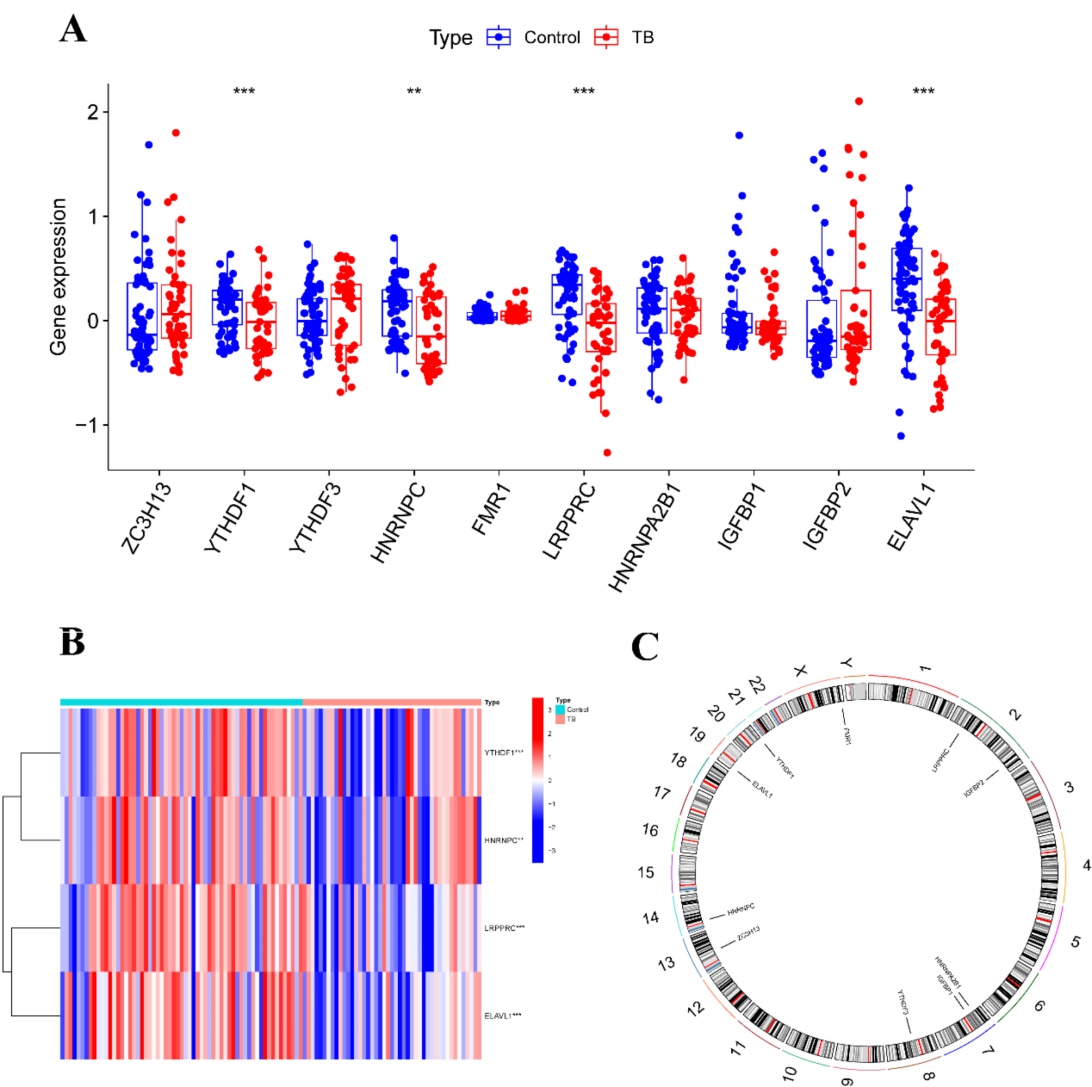
Screening of Differentially Expressed m6A Genes in Tuberculosis. (A) Boxplot of the expression levels of 10 m6A-related genes between the control and TB groups. (B) Heatmap showing the expression of 4 differentially expressed genes between the control and TB groups. (C) Chromosomal localization of m6A-related genes. *p < 0.05, **p < 0.01, ***p < 0.001.

We identified 10 m6A-targeted genes significantly associated with tuberculosis (TB) through a comprehensive differential analysis of the GSE83456 dataset, comparing TB patients with healthy controls. Notably, YTHDF1, HNRNPC, LRPPRC, and ELAVL1 have not previously been reported to be significantly associated with TB. This group included one writer gene (ZC3H13) and nine reader genes (YTHDF1, YTHDF3, HNRNPC, FMR1, LRPPRC, HNRNPA2B1, IGFBP1, IGFBP2, and ELAVL1). Among them, YTHDF1, HNRNPC, LRPPRC, and ELAVL1 showed statistical significance (*p* < 0.05) in TB patients compared with controls (Fig. 2A, B), suggesting a close association with TB progression. Additionally, the chromosomal locations of these 10 m6A-related genes were mapped to provide genomic context (Fig. 2C).

### Selection of key m6A regulatory genes using machine learning

**Fig. 3:**
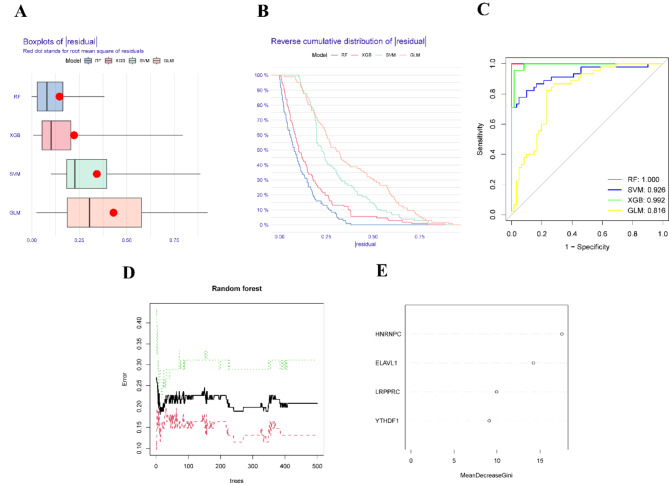
Selection of Key m6A Regulatory Genes Using Machine Learning. (**A**) Boxplot of residuals for the four machine learning algorithms. (**B**) Reverse cumulative distribution of residuals for the four machine learning algorithms. (**C**) ROC curves for the four machine learning models. (**D**) Selection of key m6A regulatory genes using the Random Forest (RF) algorithm. (**E**) Importance scores of the key m6A regulatory genes.

To refine our analysis, we employed four machine learning algorithms—Random Forest (RF), Support Vector Machine (SVM), Extreme Gradient Boosting (XGB), and Generalized Linear Model (GLM)—to identify key m6A regulatory genes associated with TB. Comparative analysis of residual values and reverse cumulative distribution curves revealed that the RF algorithm outperformed the others, demonstrating superior predictive accuracy (Fig. 3A, B). This led us to adopt the RF model as the optimal approach. ROC curve analysis further confirmed the RF model’s robustness, with the highest AUC value among the four algorithms (Fig. 3C). Subsequently, the RF model was used to rank genes by importance, identifying YTHDF1, HNRNPC, LRPPRC, and ELAVL1 as key regulatory genes based on their importance scores, all exceeding a threshold of 5 (Fig. 3D-E).

### Construction of a clinical prediction model

**Fig. 4:**
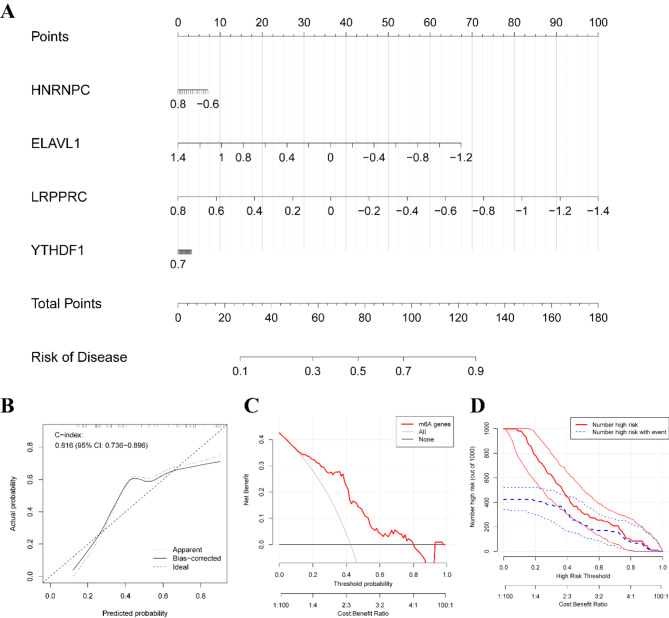
Construction of the Clinical Prediction Model. (**A**) Nomogram based on the four m6A regulatory genes. (**B**) Calibration curve evaluating the accuracy of the Nomogram model. (**C**) Decision curve for the clinical prediction model. (**D**) Clinical benefit curve for the prediction model.

Building on the identified key m6A regulatory genes, we developed a clinical prediction model using the “lrm” function from the “rms” package in R. This model, designed to estimate the likelihood of TB infection, demonstrated strong predictive performance with a C-index of 0.816, indicating high accuracy (Fig. 4A-B). Decision curve analysis (DCA) further validated the clinical utility of this model, suggesting that decisions informed by the model could offer significant benefits to TB patients (Fig. 4C). Additionally, the clinical impact curve substantiated the model’s predictive strength (Fig. 4D).

### Subtype classification of TB patients based on key m6A regulatory genes

**Fig. 5:**
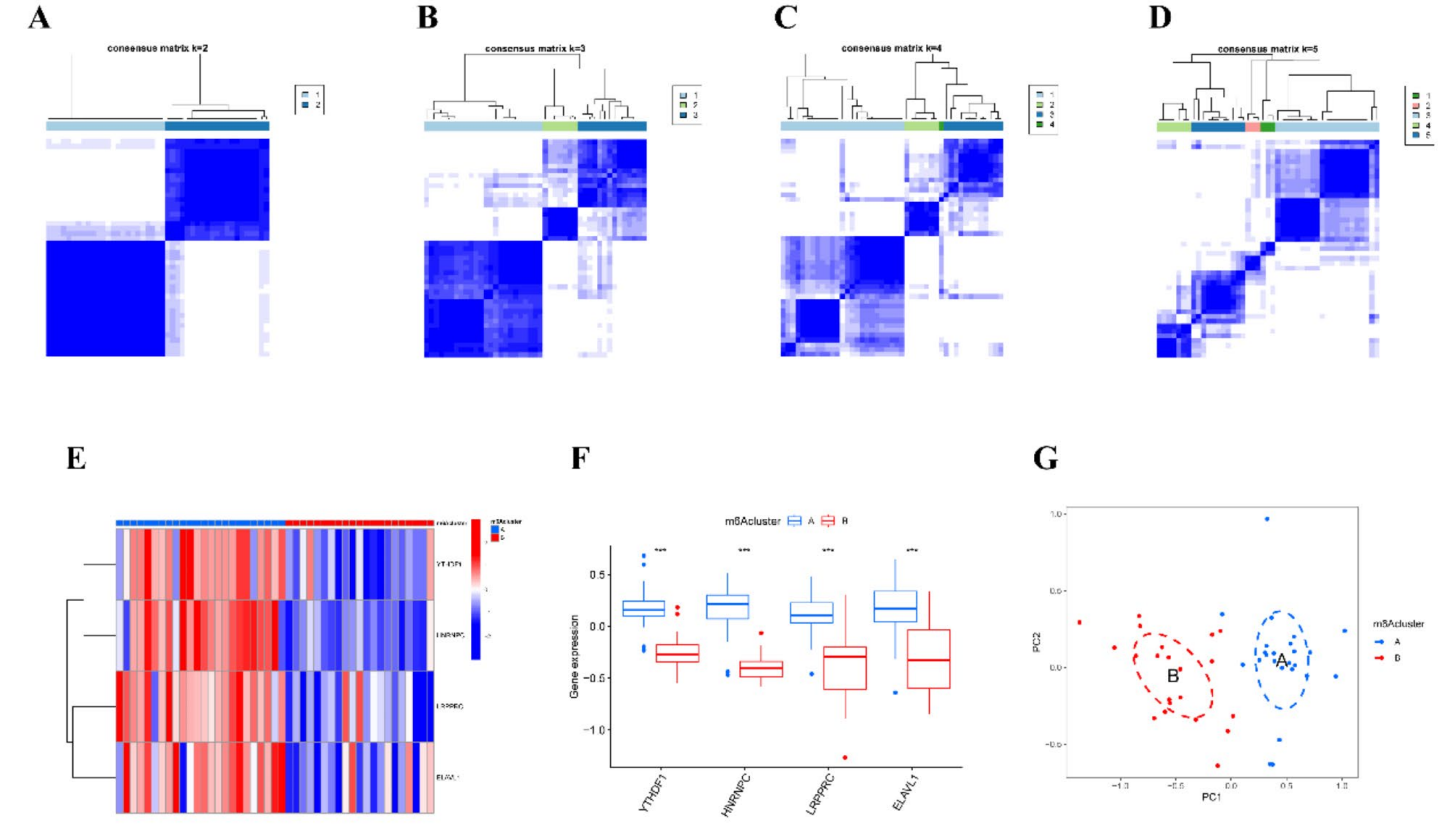
Clustering of TB Patients Based on Key m6A Regulatory Genes. (**A**-**D**) Consensus clustering of TB samples for k = 2 to k = 5. (**E**) Heatmap of the expression of four key m6A regulatory genes in cluster A and cluster B. (**F**) Boxplot showing the differential expression of the four key m6A regulatory genes in cluster A and cluster B. (**G**) PCA analysis between clusters. *p < 0.05, **p < 0.01, ***p < 0.001.

Using the four key m6A regulatory genes, we conducted a clustering analysis, which classified TB patients into two distinct m6A subtypes: cluster A and cluster B (Fig. 5A). Subsequent differential expression analysis revealed that YTHDF1, HNRNPC, LRPPRC, and ELAVL1 were expressed at higher levels in cluster A compared to cluster B (Fig. 5B-C). Principal component analysis (PCA) provided further validation, demonstrating that these four genes effectively distinguished between the TB subtypes, thereby confirming the reliability of the m6A-based classification (Fig. 5D-G).

**Fig. 6:**
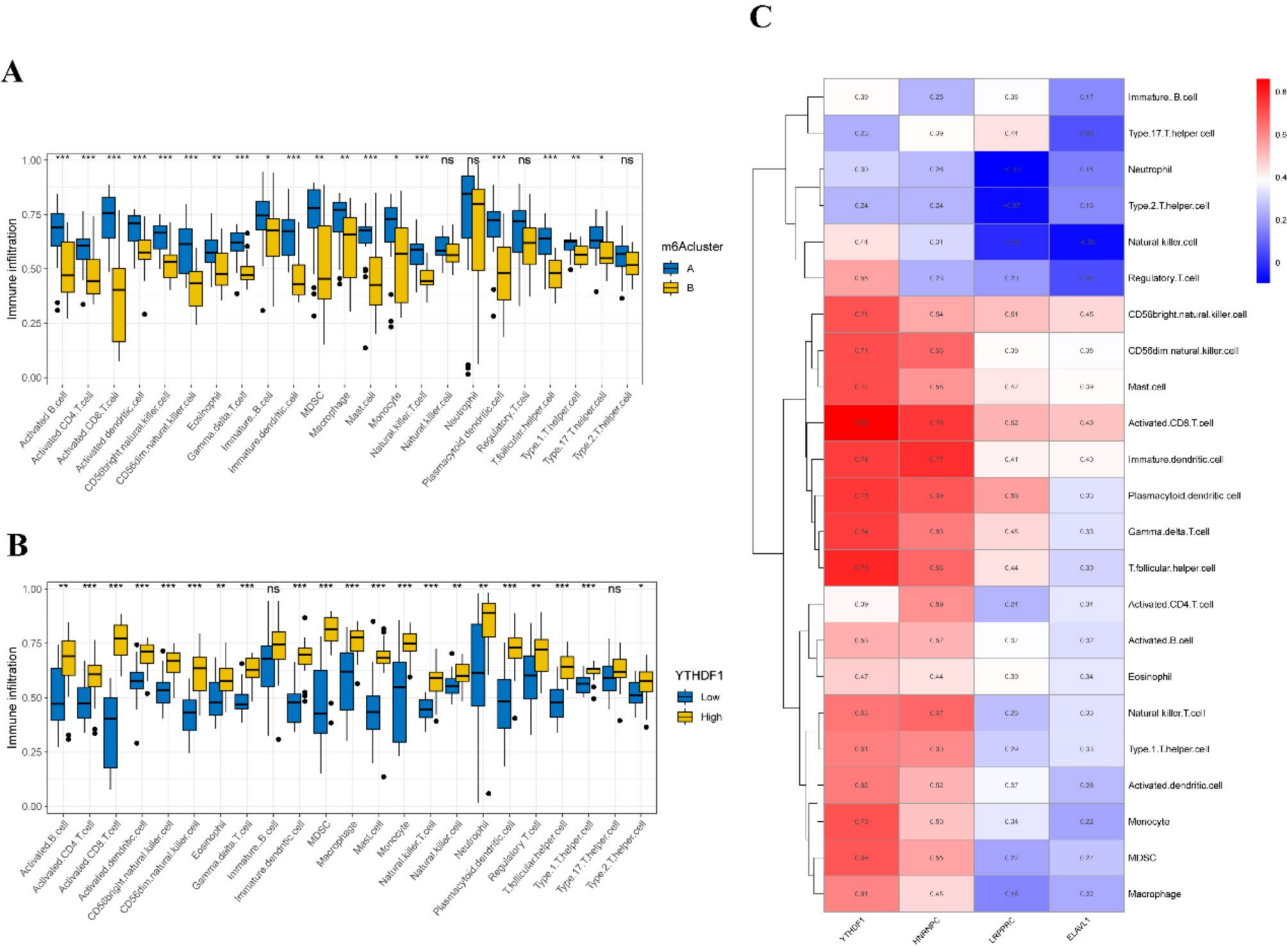
ssGSEA Enrichment Analysis. (**A**) Differences in immune cell infiltration between cluster A and cluster B. (**B**) Differences in immune cell infiltration between high and low YTHDF1 expression groups. (**C**) Correlation between the four key m6A regulatory genes and immune cell infiltration. ns > 0.05, *p < 0.05, **p < 0.01, ***p < 0.001.

To investigate the immunologic impact of these subtypes, we used ssGSEA to assess immune cell infiltration across TB subtypes. The analysis revealed significant differences in the infiltration of 19 immune cell types between the m6A subtypes (Fig. 6A). Notably, the expression of YTHDF1, HNRNPC, LRPPRC, and ELAVL1 was positively correlated with increased immune cell infiltration, suggesting a potential link between m6A regulation and immune responses in TB (Fig. 6B–C, SF1).

### Identification of differential genes between m6A subtypes and TB subtype classification

**Fig. 7:**
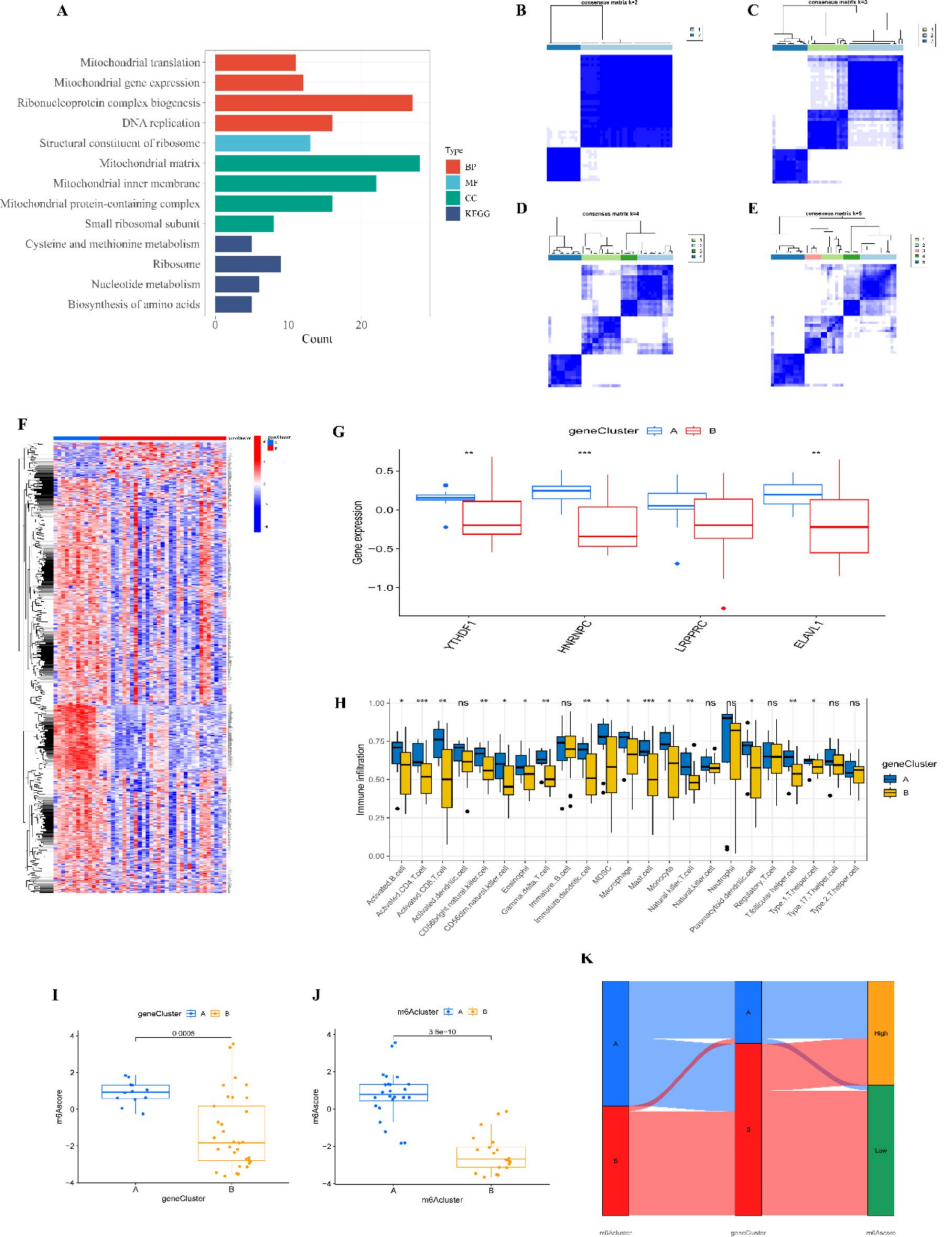
Clustering of TB Patients Based on Differential Genes Between m6A Subtypes. (**A**) GO and KEGG enrichment analyses. (**B**-**E**) Consensus clustering of differential genes for k = 2 to k = 5. (**F**) Heatmap of the expression levels of differential genes between genomic subtypes. (**G**) Boxplot showing the differential expression of key m6A regulatory genes between genomic subtypes. (**H**) Differences in immune cell infiltration between genomic subtypes. (**I**) Differences in m6A scores between genomic subtypes. (**J**) Differences in m6A scores between m6A subtypes. (**K**) Sankey diagram showing the relationships among m6A scores, m6A subtypes, and genomic subtypes. ns > 0.05, *p < 0.05, **p < 0.01, ***p < 0.001.

To further corroborate the accuracy of our m6A-based TB classification, we conducted differential gene expression analysis between the two subtypes. A total of 610 differential genes were identified, and subsequent GO and KEGG enrichment analyses revealed that these genes were predominantly associated with mitochondrial and ribosomal functions within the biological process (BP) and molecular function (MF) categories and were primarily localized to mitochondria and ribosomes in the cellular component (CC) category (Fig. 7A). KEGG analysis highlighted enrichment in mitochondrial and nucleotide metabolism pathways. Utilizing these 610 differential genes, we employed consensus clustering to classify TB patients into genomic subtypes, which aligned with the m6A subtypes, further validating the classification (Fig. 7B-E). The expression patterns of these 610 genes were visualized across the genomic subtypes, demonstrating consistent differences that mirrored those observed in m6A subtypes (Fig. 7F-H).

Finally, PCA-based calculation of m6A scores revealed that both m6A subtype A and genomic subtype A exhibited higher m6A scores compared to their respective B counterparts (Fig. 7I-J). A Sankey diagram was generated to visualize the intricate relationships between m6A scores, m6A subtypes, and genomic subtypes, offering a comprehensive overview of the classification’s accuracy and relevance (Fig. 7K).

### RT-qPCR validation of m6A key regulatory genes

**Fig. 8:**
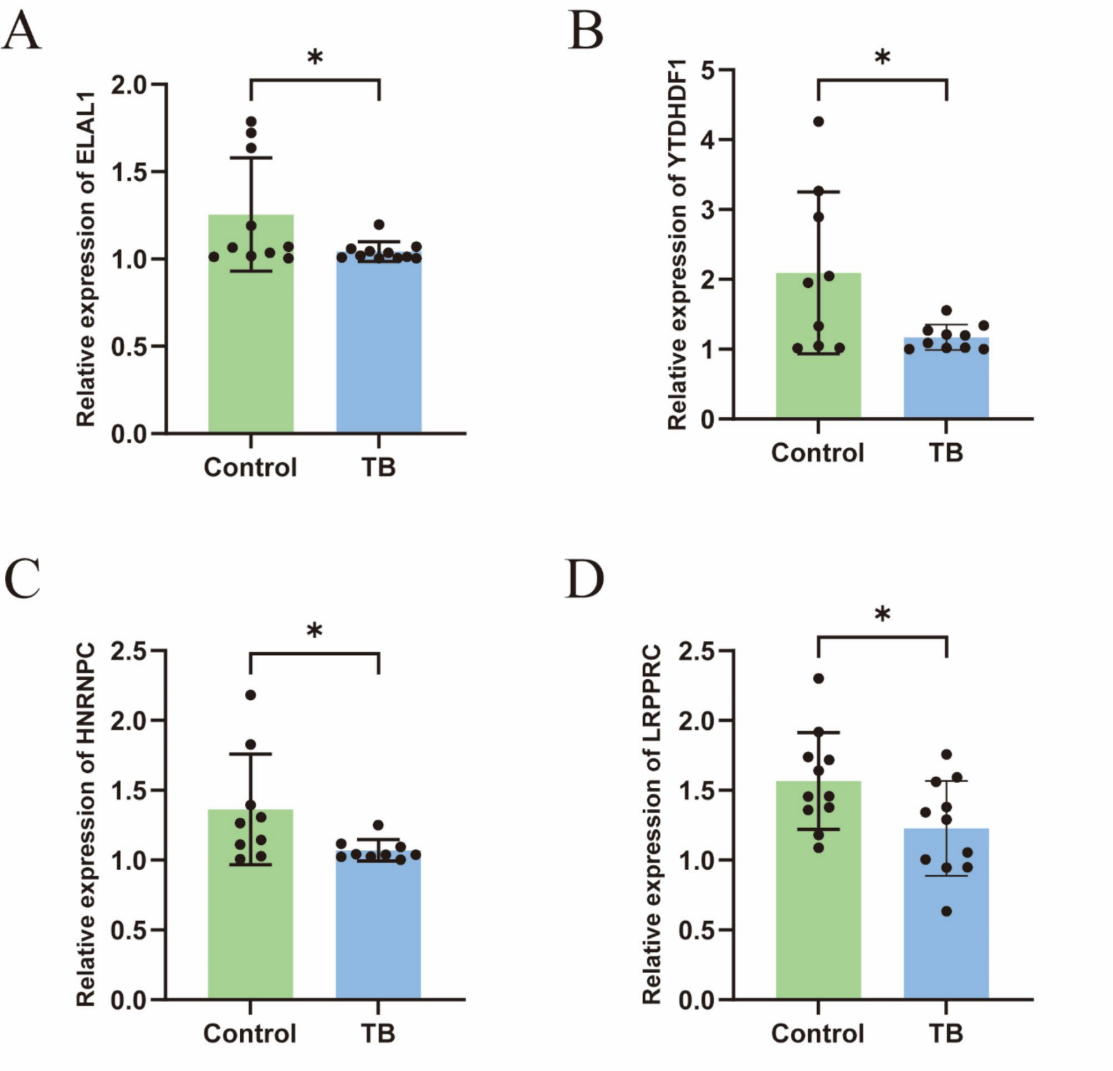
m6A-related gene expression validation. (**A**) ELAL1 gene expression validation; (**B**) YTDHDF1 gene expression validation; (**C**) HNRNPC gene expression validation; (**D**) LRPPRC gene expression validation.

We conducted RT-qPCR experiments to verify the expression levels of key m6A regulatory genes. As shown in Fig. 8, the expression levels of YTHDF1, HNRNPC, LRPPRC, and ELAVL1 in the TB group were significantly lower than in the control group, consistent with the results of the bioinformatics analysis.

## Discussion

The pathogenesis of tuberculosis (TB) is characterized by a critical feature: the ability of Mycobacterium tuberculosis (MTB) to survive and persist within diverse intracellular environments, particularly within various myeloid cell populations^16,17^. The initial phase of MTB infection is often clinically silent, manifesting primarily at the molecular and genetic levels. Despite this latent stage, the infection poses a significant threat to patient health. Recent research has underscored the importance of RNA N6-methyladenosine (m6A) modification in regulating the onset and progression of TB^5,18,19^. However, the specific mechanisms by which m6A regulatory genes contribute to TB pathophysiology remain largely unexplored. In this study, we systematically revealed for the first time the specific regulatory mechanism of m6A modification in the development and progression of tuberculosis (TB). Our results indicate that m6A modification is not only involved in the regulation of TB-related gene expression, but also influences the pathological process of TB by modulating the immune microenvironment. This finding fills a gap in the existing literature and provides a new molecular perspective on the immune regulation of TB. Unlike previous m6A studies that focused mainly on cancer and viral infections, our innovative findings in bacterial infections, especially Mycobacterium tuberculosis infection, further expand the application prospects of m6A modification^20,21^.

Our study began with a differential analysis between TB patients and healthy controls, identifying four significant m6A regulatory genes—YTHDF1, HNRNPC, LRPPRC, and ELAVL1—from an initial panel of 10 candidates. Notably, these genes were significantly downregulated in TB patients. YTHDF1, for example, encodes an RNA-binding protein that promotes mRNA translation by interacting with the m6A motif within the 5’ UTR^22,23^. In contrast to YTHDF2, YTHDF1 binds earlier in the mRNA lifecycle, exerting its influence at a more foundational stage^24^. Previous studies have shown that YTHDF1 enhances the expression of WW domain-containing E3 ubiquitin protein ligase 1 (WWP1), a critical component of the ubiquitin-proteasome pathway, which is implicated in various diseases, including infectious conditions^25,26^. In the context of TB, our findings suggest that YTHDF1 downregulation could impair WWP1 expression, thereby weakening the host’s ability to regulate protein degradation processes essential for immune defense. This reduced expression could, in turn, facilitate the survival and replication of MTB within host cells. Our results, consistent with previous research, indicate that YTHDF1 mRNA expression is markedly lower in TB patients compared to healthy controls^18^.

In addition to YTHDF1, HNRNPC has been implicated in promoting apoptosis and necrosis by mediating ATF4 m6A modification, potentially serving as a mechanism through which MTB evades host immune surveillance^27^. HNRNPC facilitates the m6A modification of ATF4, a key factor in cellular stress responses and apoptotic pathways^28^. In the context of MTB infection, this regulation may lead to increased host cell death, thereby disrupting immune defense mechanisms and allowing MTB to evade immune detection^28–30^. By inducing apoptosis, HNRNPC may create a stable intracellular environment conducive to bacterial survival, ultimately contributing to TB pathogenesis. LRPPRC, a regulator of mRNA encoded by mitochondrial DNA, plays a significant role in viral infections by inhibiting antiviral signaling mediated by the mitochondrial antiviral-signaling protein (MAVS), acting as a suppressor in hepatitis C virus infection^31^. However, its role in bacterial infections, particularly with MTB, remains to be fully elucidated. Our study suggests that LRPPRC might modulate immune signaling pathways similarly during MTB infection, potentially inhibiting MAVS-mediated antiviral responses essential for controlling intracellular pathogens. This suppression of mitochondrial antiviral signaling could impair host immunity against MTB, enabling the bacteria to persist within host cells. Although further experimental validation is necessary, our data indicate that LRPPRC downregulation may contribute to weakened immune responses in TB patients. ELAVL1, another key gene, is instrumental in angiogenesis, apoptosis, and inflammation, functioning in both pro-inflammatory and anti-inflammatory processes through the regulation of PARP1^32,33^. In TB patients, our findings suggest that ELAVL1 downregulation may alter inflammatory responses within the immune microenvironment. By regulating PARP1, ELAVL1 influences processes such as DNA repair and apoptosis, which are crucial for maintaining immune homeostasis. Reduced ELAVL1 expression may weaken inflammatory responses against MTB, impairing the host’s ability to effectively counteract infection. Collectively, these four m6A regulatory genes—YTHDF1, HNRNPC, LRPPRC, and ELAVL1—appear to play pivotal roles in the pathogenesis and progression of TB by modulating key immune and inflammatory pathways.

To enhance the identification of key m6A-regulated genes, we employed four machine learning algorithms and concluded that the Random Forest (RF) algorithm was the most effective, as demonstrated by its superior residual values and high AUC in ROC curve analysis. The RF algorithm identified YTHDF1, HNRNPC, LRPPRC, and ELAVL1 as the most critical genes involved in TB pathogenesis. Our findings regarding these novel m6A target genes may have significant clinical applications in TB. For instance, these m6A-modified genes could serve as early diagnostic markers for TB, offering a reliable basis for patient staging and prognosis assessment. By modulating the m6A modification levels of YTHDF1, HNRNPC, LRPPRC, and ELAVL1, it may be possible to enhance immune responses against MTB, providing a novel therapeutic approach to control disease progression. These insights not only highlight the potential of m6A modification as a diagnostic and therapeutic target in TB but also pave the way for future research into the molecular mechanisms of immune regulation in bacterial infections.

Furthermore, m6A modification-related genes might represent new therapeutic targets. By modulating the m6A modification levels of these genes, it may be possible to enhance patient immune responses and control disease progression. Additionally, we developed a clinical prediction model that enables clinicians to estimate the likelihood of TB infection by evaluating the expression levels of these four genes. This model enhances TB diagnostic accuracy, providing a theoretical foundation and experimental support for the future development of m6A-based diagnostic and therapeutic tools.

Grouping patients based on disease-related biomarkers is a well-established approach in clinical practice. For instance, a study demonstrated that stratifying Parkinson’s disease patients using serum biomarkers effectively predicted motor and non-motor outcomes, thereby guiding early clinical intervention^34^. In our consensus clustering analysis based on key m6A genes, we classified TB patients into two subtypes (Cluster A and Cluster B) and observed significant differences in immune cell infiltration characteristics between the subtypes. Specifically, Cluster A exhibited significantly higher levels of B-cell, NK-cell, monocyte, macrophage, and T-cell infiltration, whereas these levels were notably reduced in Cluster B (*p* < 0.05). This finding suggests two distinct modes of immune response: the high immunoreactivity of Cluster A may support host control of Mycobacterium tuberculosis, while the low immune infiltration in Cluster B may indicate a greater ability of the pathogen to evade immune surveillance.

The differences in immune cell composition between the high immune infiltration subtype (Cluster A) and the low immune infiltration subtype (Cluster B) underscore the potential clinical significance of these subtypes. For example, patients in Cluster A may respond more effectively to immune-enhancing therapies, whereas those in Cluster B may require additional immune activation strategies to boost their immune responses. This subtype differentiation has potential implications for the development of future personalized therapies. Furthermore, immune subtype-based stratification in other diseases (e.g., cancer and infectious diseases) has proven valuable in identifying how different immune microenvironments influence disease progression^]^. We propose that future studies should further explore the specific immune responses associated with TB subtypes by characterizing these subtypes in larger samples or independent datasets. Such studies could provide a theoretical foundation for developing personalized therapeutic strategies.

Mycobacterium tuberculosis is known to infect macrophages, where it survives and proliferates, thereby driving TB progression^35^. Extensive research underscores the critical role of macrophages in TB infection and granuloma formation, positioning them as key players in disease progression^3^. Eosinophils, among the first responders during MTB infection, interact with macrophages to maintain tissue homeostasis and sustain resident macrophages during infection^36^. Furthermore, Th1 cells have been shown to prevent TB by producing interferon-gamma (IFN-γ) and stimulating anti-MTB responses within macrophages^37,38^. These findings collectively suggest that m6A regulatory genes may modulate TB progression by influencing inflammatory and immune processes.

To corroborate the accuracy of our m6A-based classification, we identified two genomic subtypes of TB through differential gene analysis. The m6A scores, calculated via PCA, provided a quantitative reference for assessing these subtypes’ characteristics, offering valuable insights for future clinical diagnostics and treatment strategies. Additionally, we validated the expression levels of YTHDF1, HNRNPC, LRPPRC, and ELAVL1 in TB patients through real-time quantitative PCR (RT-qPCR), confirming significant downregulation in TB patients compared to controls (Fig. 8). These findings further underscore the role of m6A regulatory genes in TB progression, contributing to a deeper understanding of the disease’s molecular mechanisms.

Although this study highlights the critical role of m6A modifications in TB immunomodulation, several key questions remain unresolved. For example, the specific functions of individual m6A-modifying enzymes in Mycobacterium tuberculosis are still unclear, and the effects of m6A modifications on immune cell migration and drug resistance warrant further investigation. Future studies could employ knockdown or overexpression experiments to elucidate how these m6A modification genes influence TB progression, thereby providing stronger evidence for molecular diagnostics and personalized treatment strategies.

This study also has certain limitations related to technology and data sources. First, as the primary data source, the GEO database may introduce bias due to limitations in sample size, sampling conditions, and geographical constraints, potentially affecting the external validity of our findings. Additionally, variability in sample handling and collection methods may impact data consistency. Although we applied data standardization and multiple quality control steps to mitigate these biases, future studies should integrate multicenter, large-scale datasets to better validate the robustness and applicability of our results.

Another limitation is the relatively small sample size in the validation experiment, which may compromise the robustness of the statistical findings. Future studies should increase sample sizes to improve the consistent performance of m6A-regulated genes across diverse populations, thereby enhancing the external validity of the conclusions. Larger sample sizes would also provide more reliable data for examining the functional roles of these genes in patients with different clinical subtypes of TB, thus laying a stronger foundation for clinical applications. A large-scale, multicenter study is anticipated to further substantiate our findings and support the potential application of m6A-regulated genes in TB diagnosis and treatment.

## Conclusion

In this study, we systematically elucidated the dynamic regulatory features of m6A modification across different pathological stages of tuberculosis (TB). We identified a set of m6A target genes and explored their potential roles in modulating the TB immune microenvironment. These findings address existing knowledge gaps in TB research and suggest possible avenues for the future development of TB diagnostic markers and targeted therapies that leverage m6A modification. Our results indicate that m6A modification may act as an important regulator of TB onset and progression, providing a basis for further investigation into early TB diagnosis and the potential design of personalized therapeutic strategies. This study offers insights that could inform future clinical research and practice.

## Electronic supplementary material

Below is the link to the electronic supplementary material.

Supplementary Material 1

## Data Availability

The direct links required to find each data set in the database are as follows: the GEO gene expression and clinical pathology data set: https://www.ncbi.nlm.nih.gov/geo/query/acc.cgi?acc=GSE83456. The data set downloaded by this direct link is the original data set.

## Abbreviations

TB: Tuberculosis
RT-qPCR: Real-time quantitative PCR
GEO: Gene Expression Omnibus
GSEA: Gene Set Enrichment Analysis
m6A: N6-methyladenosine
MTB: Mycobacterium tuberculosis

## References

[1] Furin, J., Cox, H. & Pai, M. Tuberculosis *Lancet*, **393**(10181):1642–1656. (2019). 30904262 10.1016/S0140-6736(19)30308-3

[2] Sheng, Y., Hua, H., Yong, Y. & Zhou, L. Identification of hub genes and typing of tuberculosis infections based on autophagy-related genes. *Pol. J. Microbiol.* **72** (3), 223–238 (2023). 37725899 10.33073/pjm-2023-022 PMC10561080

[3] Wufuer, D., Li, Y., Aierken, H. & Zheng, J. Bioinformatics-led discovery of ferroptosis-associated diagnostic biomarkers and molecule subtypes for tuberculosis patients[J]. *Eur. J. Med. Res.* **28** (1), 445 (2023). 37853432 10.1186/s40001-023-01371-5 PMC10585777

[4] Jiang, L. et al. Folic acid protects against isoniazid-induced liver injury via the m6A RNA methylation of cytochrome P450 2E1 in mice[J]. *Front. Nutr.* **11**, 1389684 (2024). 38798770 10.3389/fnut.2024.1389684 PMC11116731

[5] Zhang, T. P., Li, R., Wang, L. J., Huang, Q. & Li, H. M. Roles of the m6A methyltransferases METTL3, METTL14, and WTAP in pulmonary tuberculosis[J]. *Front. Immunol.* **13**, 992628 (2022). 36569923 10.3389/fimmu.2022.992628 PMC9768477

[6] Huang, Z. et al. Circulating circular RNAs hsa_circ_0001204 and hsa_circ_0001747 act as diagnostic biomarkers for active tuberculosis detection. *Int. J. Clin. Exp. Pathol.* **11** (2), 586–594 (2018). 31938144 PMC6958007

[7] Wang, X. et al. N(6)-methyl adenosine modulates Messenger RNA translation efficiency. *Cell* **161** (6), 1388–1399 (2015). cell.2015.05.014. 26046440 10.1016/j.cell.2015.05.014 PMC4825696

[8] Gu, C. et al. RNA m6A modification in cancers: Molecular mechanisms and potential clinical applications. *Innov. (Camb)*. **1** (3), 100066 (2020). 10.1016/j.xinn.2020.100066 PMC8454620 34557726

[9] Huang, H., Weng, H. & Chen, J. m6A modification in Coding and non-coding RNAs: roles and therapeutic implications in Cancer. *Cancer Cell.* **37** (3), 270–288. 10.1016/j.ccell.2020.02.004 (2020). PMID: 32183948; PMCID: PMC7141420. 32183948 10.1016/j.ccell.2020.02.004 PMC7141420

[10] Pan, J., Huang, T., Deng, Z. & Zou, C. Roles and therapeutic implications of m6A modification in cancer immunotherapy. *Front. Immunol.* **14**, 1132601. 10.3389/fimmu.2023.1132601 (2023). PMID: 36960074; PMCID: PMC10028070. 36960074 10.3389/fimmu.2023.1132601 PMC10028070

[11] Cao, L., Huang, G., Fan, J., Liu, X. & Ma, Z. Role of N6-methyladenosine methylation in head and neck cancer and its regulation of innate immune pathways. *Front. Immunol.* **15**, 1458884. 10.3389/fimmu.2024.1458884 (2024). PMID: 39403369; PMCID: PMC11471572. 39403369 10.3389/fimmu.2024.1458884 PMC11471572

[12] Shen, L. & Yue, S. M6A-related bioinformatics analysis indicates that LRPPRC is an immune marker for ischemic stroke[J]. *Sci. Rep.* **14** (1), 8852 (2024). 38632288 10.1038/s41598-024-57507-y PMC11024132

[13] Renshaw, P. S. et al. Conclusive evidence that the major T-cell antigens of the Mycobacterium tuberculosis complex ESAT-6 and CFP-10 form a tight, 1:1 complex and characterization of the structural properties of ESAT-6, CFP-10, and the ESAT-6*CFP-10 complex. Implications for pathogenesis and virulence[J]. *J. Biol. Chem.* **277** (24), 21598–21603 (2002). 11940590 10.1074/jbc.M201625200

[14] Ma, M. et al. Mycobacterium tuberculosis inhibits METTL14-mediated m6A methylation of Nox2 mRNA and suppresses anti-TB immunity[J]. *Cell. Discov*. **10** (1), 36 (2024). 38548762 10.1038/s41421-024-00653-4 PMC10978938

[15] Kanehisa, M. & Goto, S. KEGG: kyoto encyclopedia of genes and genomes. *Nucleic Acids Res.* **28** (1), 27–30. 10.1093/nar/28.1.27 (2000). PMID: 10592173; PMCID: PMC102409. 10592173 10.1093/nar/28.1.27 PMC102409

[16] Blankley, S. et al. The Transcriptional signature of active tuberculosis reflects Symptom Status in Extra-pulmonary and Pulmonary Tuberculosis[J]. *PLoS One*. **11** (10), e0162220 (2016). 27706152 10.1371/journal.pone.0162220 PMC5051928

[17] Chandra, P., Grigsby, S. J. & Philips, J. A. Immune evasion and provocation by Mycobacterium tuberculosis[J]. *Nat. Rev. Microbiol.* **20** (12), 750–766 (2022). 35879556 10.1038/s41579-022-00763-4 PMC9310001

[18] Li, H. M. et al. Association of N6-methyladenosine readers’ genes variation and expression level with pulmonary tuberculosis[J]. *Front. Public. Health*. **10**, 925303 (2022). 36072379 10.3389/fpubh.2022.925303 PMC9441624

[19] Zhang, T. P., Li, R., Wang, L. J. & Li, H. M. Impact of m6A demethylase (ALKBH5, FTO) genetic polymorphism and expression levels on the development of pulmonary tuberculosis[J]. *Front. Cell. Infect. Microbiol.* **12**, 1074380 (2022). 36619747 10.3389/fcimb.2022.1074380 PMC9817133

[20] Wu, C. et al. Interplay of m6A and H3K27 trimethylation restrains inflammation during bacterial infection. *Sci. Adv.* **6** (34), eaba0647. 10.1126/sciadv.aba0647 (2020). PMID: 32875102; PMCID: PMC7438091. 32875102 10.1126/sciadv.aba0647 PMC7438091

[21] Lu, Q. et al. Mycobacterium tuberculosis Rv1096, facilitates mycobacterial survival by modulating the NF-κB/MAPK pathway as peptidoglycan N-deacetylase. Mol Immunol. ;127:47–55. doi: 10.1016/j.molimm.2020.08.005. Epub 2020 Sep 11. PMID: 32927163. (2020). 10.1016/j.molimm.2020.08.005 32927163

[22] Zhuang, M. et al. The m6A reader YTHDF1 regulates axon guidance through translational control of Robo3.1 expression[J]. *Nucleic Acids Res.* **47** (9), 4765–4777 (2019). 30843071 10.1093/nar/gkz157 PMC6511866

[23] Xia, H. et al. N6-Methyladenosine-modified circSAV1 triggers ferroptosis in COPD through recruiting YTHDF1 to facilitate the translation of IREB2[J]. *Cell. Death Differ.* **30** (5), 1293–1304 (2023). 36828914 10.1038/s41418-023-01138-9 PMC10154389

[24] Rong, H., Wang, D., Wang, Y., Dong, C. & Wang, G. YTHDF1 in Tumor Cell metabolism: an updated Review[J]. *Molecules* **29** (1), 140 (2023). 38202722 10.3390/molecules29010140 PMC10779796

[25] Zhang, S. et al. YTHDF1 alleviates sepsis by upregulating WWP1 to induce NLRP3 ubiquitination and inhibit caspase-1-dependent pyroptosis[J]. *Cell. Death Discov*. **8** (1), 244 (2022). 35508474 10.1038/s41420-022-00872-2 PMC9068740

[26] Zhi, X. & Chen, C. WWP1: a versatile ubiquitin E3 ligase in signaling and diseases[J]. *Cell. Mol. Life Sci.* **69** (9), 1425–1434 (2012). 22051607 10.1007/s00018-011-0871-7 PMC11114891

[27] Mo, K. et al. Targeting hnRNPC suppresses thyroid follicular epithelial cell apoptosis and necroptosis through m6A-modified ATF4 in autoimmune thyroid disease[J]. *Pharmacol. Res.* **196**, 106933 (2023). 37729957 10.1016/j.phrs.2023.106933

[28] Liu, N. et al. N(6)-methyladenosine-dependent RNA structural switches regulate RNA-protein interactions. *Nature* **518** (7540), 560–564 (2015). 25719671 10.1038/nature14234 PMC4355918

[29] Wang, L. C. et al. M6A RNA methylation Regulator HNRNPC contributes to Tumorigenesis and predicts prognosis in Glioblastoma Multiforme. *Front. Oncol.* **8**, 10:536875 (2020 Oct). 10.3389/fonc.2020.536875 PMC7578363 33134160

[30] Zhao, Y., Shi, Y., Shen, H. & Xie, W. m6A-binding proteins: the emerging crucial performers in epigenetics. *J. Hematol. Oncol.* **13** (1), 35 (2020). 32276589 10.1186/s13045-020-00872-8 PMC7146974

[31] Cui, J., Wang, L., Ren, X., Zhang, Y. & Zhang, H. LRPPRC: a multifunctional protein involved in Energy Metabolism and Human Disease[J]. *Front. Physiol.* **10**, 595 (2019). 31178748 10.3389/fphys.2019.00595 PMC6543908

[32] Chen, H. Y. et al. ELAVL1 is transcriptionally activated by FOXC1 and promotes ferroptosis in myocardial ischemia/reperfusion injury by regulating autophagy[J]. *Mol. Med.* **27** (1), 14 (2021). 33568052 10.1186/s10020-021-00271-w PMC7874472

[33] Tanwar, V. S. et al. Palmitic Acid-Induced Long Noncoding RNA PARAIL regulates inflammation via Interaction with RNA-Binding protein ELAVL1 in Monocytes and Macrophages[J]. *Arterioscler. Thromb. Vasc Biol.* **43** (7), 1157–1175 (2023). 37128912 10.1161/ATVBAHA.122.318536 PMC10287039

[34] Lawton, M. et al. Blood biomarkers with Parkinson’s disease clusters and prognosis: the oxford discovery cohort[J]. *Mov. Disord*. **35** (2), 279–287 (2020). 31693246 10.1002/mds.27888 PMC7028059

[35] Li, S. et al. Identification of immune infiltration and cuproptosis-related molecular clusters in tuberculosis[J]. *Front. Immunol.* **14**, 1205741 (2023). 37497230 10.3389/fimmu.2023.1205741 PMC10366538

[36] Bohrer, A. C. et al. Rapid GPR183-mediated recruitment of eosinophils to the lung after Mycobacterium tuberculosis infection[J]. *Cell. Rep.* **40** (4), 111144 (2022). 35905725 10.1016/j.celrep.2022.111144 PMC9460869

[37] Lyadova, I. V., Panteleev, A. V. & Hwang, S. Th1 and Th17 cells in tuberculosis: Protection, Pathology, and Biomarkers[J]. *Mediat. Inflamm.* **2015**, 854507–854513 (2015). 10.1155/2015/854507 PMC4657112 26640327

[38] Jasenosky, L. D., Scriba, T. J., Hanekom, W. A. & Goldfeld, A. E. T cells and adaptive immunity to Mycobacterium tuberculosis in humans[J]. *Immunol. Rev.* **264** (1), 74–87 (2015). 25703553 10.1111/imr.12274

