# Supplementary figures and images for: Identification of diagnostic biomarkers and molecular subtype analysis associated with m6A in Tuberculosis immunopathology using machine learning
Source: Sci Rep. 2024 Dec 2;14:29982. doi: 10.1038/s41598-024-81790-4 (PMC11612281; doi:10.1038/s41598-024-81790-4)

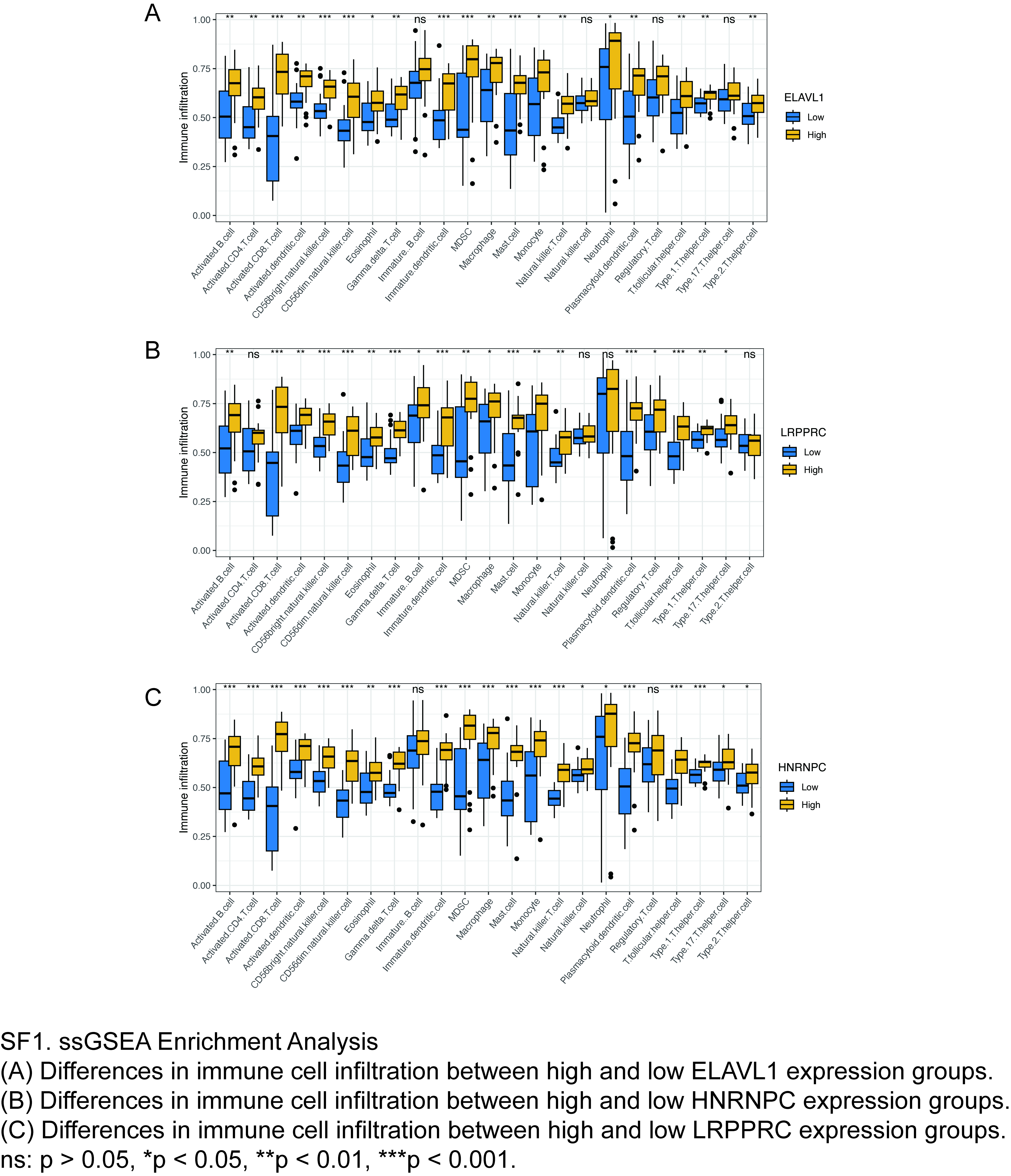

Supplement: Supplementary file 1 — Supplementary Material 1 [file 41598_2024_81790_MOESM1_ESM.tif]
